# Volume prediction for large brain metastases after hypofractionated gamma knife radiosurgery through artificial neural network

**DOI:** 10.1097/MD.0000000000030964

**Published:** 2022-10-07

**Authors:** Hyeong Cheol Moon, Young Seok Park

**Affiliations:** a Department of Neurosurgery, Gamma Knife Icon Center, Chungbuk National University Hospital, Cheongju, Cheongju, Republic of Korea; b Department of Medical Neuroscience, College of Medicine, Chungbuk National University, Cheongju, Republic of Korea; c Department of Neurosurgery, College of Medicine, Chungbuk National University, Cheongju, Republic of Korea.

**Keywords:** artificial neural network, gamma knife radiosurgery, hypofractionated, large brain metastases

## Abstract

The effectiveness of single-session gamma knife radiosurgery (GKRS) for small metastatic brain tumors has been proven, but hypofractionated GKRS (hfGKRS) for large brain metastases (BM) from the linear quadratic (LQ) model is uncertain. The purpose of this study was to investigate volume changes large BM after hfGKRS from the LQ model and predict volume changes using artificial neural network (ANN). We retrospectively investigated the clinical findings of 28 patients who underwent hfGKRS with large BM (diameter >3 cm or volume >10 cc). A total of 44 tumors were extracted from 28 patients with features. We randomly divided 30 large brain tumors as training set and 14 large brain tumors as test set. To predict the volume changes after hfGKRS, we used ANN models (single-layer perceptron (SLP) and multi-layer perceptron (MLP)). The volume reduction was 96% after hfGKRS for large BM from the LQ model. ANN model predicted volume changes with 70% and 80% accuracy for SLP and MLP, respectively. Even in large BM, hfGKRS from the LQ model could be a good treatment option. Additionally, the MLP model could predict volume changes with 80% accuracy after hfGKRS for large BM.

## 1. Introduction

Brain metastases (BM) are commonly considered intracranial tumors that are complicated by systemic cancers and are a key cause of morbidity and mortality in patients.^[1]^ The incidence of BM has increased, which could be attributed to longer survival because of better local tumor control achieved through methods such as surgery, radiotherapy, radiosurgery, and systemic chemotherapy.^[2]^ Recently, stereotactic radiosurgery (SRS) such as gamma knife radiosurgery (GKRS) has been growing in popularity as a treatment for metastatic brain tumors.^[3–5]^ However, large lesions (>diameter 3 cm) remain difficult to control with SRS due to radiation toxicity relative to single-session SRS and have been reported in some studies.^[6–9]^ Previous reports have rarely demonstrated treating large BM by GKRS.^[10,11]^ A new generation of Gamma Knife ICON^TM^ with mask fixation has the potential for fractionated treatments. Gamma Knife ICON^TM^ can detect patient movement tracking from high-definition motion management (HDMM) camera, define stereotactic references using cone-beam computed tomography (CBCT), and potentially enable hypofractionated treatment using mask fixation. Although hypofractionated GKRS (hfGKRS) treatments from the linear quadratic (LQ) model allow for the effective treatment of large BM, the optimized parameters including treatment period, dose prescription, and volume outcomes are unclear.

Recently, statistical and mathematical models have been developed for clinical decision-making, which is a key field for researchers. Models for clinical fields can help physicians in decision-making, optimizing treatment plans, and preventing the development of risk factors. Artificial neural network (ANN) and regression models have been used to predict outcomes.^[12]^ ANN is a powerful analyzer that discovers the complex and non-linear relationships between data sets and imitates the biological nervous system.^[13]^ The incorporation of ANN is one of the major challenges in developing prediction models for radiosurgery fields. Gradient-based algorithms are the most frequently trained algorithms.^[14]^

In this study, we investigated volume changes for large BM after hfGKRS from the LQ model. In addition, we predicted the volume changes using ANN.

## 2. Methods

### 2.1. Patient characteristics

We retrospectively created data to analyze patients with large BM from 2018 to 2021 at our Gamma Knife Icon Center after obtaining institute ethical clearance. A total of 28 patients (18 males and 10 females; age range 35–85 years old; median age, 69.5 years old) were previously diagnosed with large BM at Chungbuk National University Hospital. General characteristics were noted such as gender, age, pathology, recursive partitioning analysis (RPA),^[15]^ Karnofsky performance scale (KPS) score,^[16]^ and GKRS operate report. We analyzed 44 large brain lesions in 28 patients.

### 2.2. Magnetic resonance imaging acquisition

All subjects underwent magnetic resonance imaging (MRI) scans using a 1.5T MR system (Philips Achieva, Best, the Netherlands). A standard dose of gadolinium-diethylene triamine pentaacetic acid (0.1 mmol/kg body weight) was administered intravenously 10 minutes before the acquisition of contiguous three-dimensional T1-weighted enhanced images (slice thickness, 1.0 mm; repetition time/echo time, 25/46.2 milliseconds; flip angle, 30°; field of view 256 × 256 and 240 × 240 matrices; the number of sections, 80; acquisition time, 360–420 seconds). We also added T1-weighted and T2-weighted images for diagnosis. All images with motion artifacts were excluded.

### 2.3. GKRS planning for image analysis

Based on T1-weighted enhanced images, the number of large BM at referral was determined with the consent of medical physicist/neurosurgeon/radiologist trained in medical imaging and neuro-oncology. All patients were required at minimum to undergo a follow-up MRI at approximately 3 months. Tumor volume was measured through the Leksell Gamma Plan (Elekta Instrument AB, version 11.1) with manual and semi-automatic segmentation.

### 2.4. Feature extraction

Feature extraction was performed from electronic medical records and planning parameters: gender, age, KPS, RPA, shots, beam-on-time, coverage, selectivity, gradient index, prescription dose (50% margin), number of fractionation, biological equivalent dose (biologically effective dose [BED]_10_), number of lesions, diagnosis, initial tumor volume, lesion area, and tumor volume after hfGKRS. BED_10_ was investigated for the fractionated treatment effects by the LQ model.^[17,18]^ The lesion areas were defined in the major lobes (frontal, temporal, parietal, occipital, and cerebellum).

### 2.5. Artificial neural network

**Figure 1.**
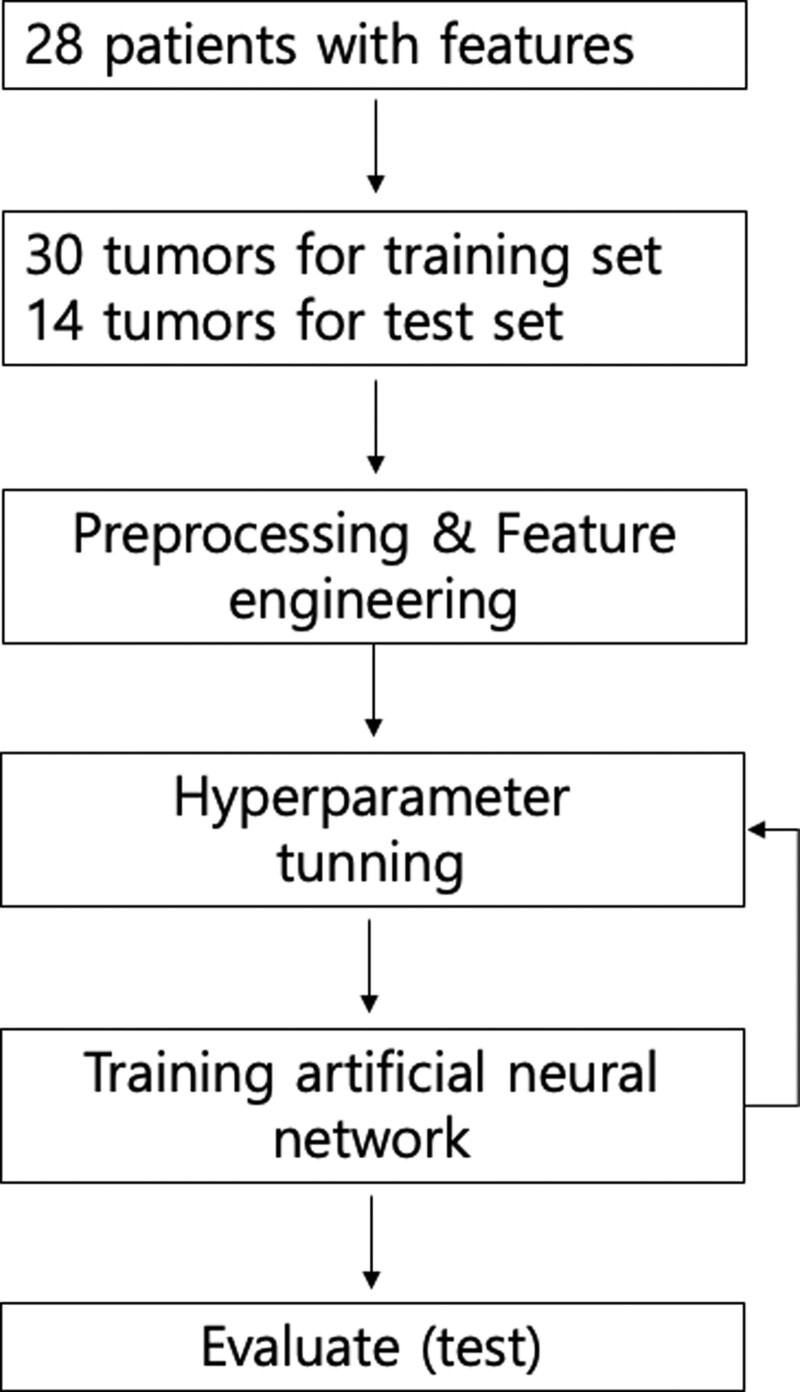
The flowchart of artificial neural network modeling for predicting volume changes.

We used the Keras (version 2.2.4), Pandas (version 0.23), and scikit-learn python (version 3.3) libraries. The single-layer perceptron (SLP) consists of a single layer of output nodes, which directly lead to the outputs via weights and bias. The multi-layer perceptron (MLP) consists of feed-forward algorithms, which are known in many applications as the functional approximation, and a back-propagation network is used for training. Our training process was performed with Keras tools for categorical cross-entropy loss and with stochastic gradient descent for optimization. A learning rate of 1.0 × 10^-6^. The flowchart of ANN modeling was shown in Figure 1.

## 3. Results

### 3.1. Clinical characterization

The characteristics of the 28 patients who underwent hfGKRS for large BM were listed in Table 1. The patients were followed up every 3 months following to detect tumor recurrence or newly occurring tumors. The initial tumor volume was 14.0 ± 5.3 cc and the tumor volume after hfGKRS was 9.0 ± 4.3 cc. The radiation response for large BM was shown except in two cases.

**Table 1:** Clinical characteristics and planning parameters for gamma knife radiosurgery.

Gender (male/female)		18/10
Age (median)		69.5 ± 15.7
KPS	>80	7
	≦80	21
RPA	Class 1	13
	Class 2	15
Shots		25 ± 7.8
Beam-on-time (min)		25.3 ± 6.3
Coverage (%)		0.9 ± 0.1
Selectivity (%)		0.8 ± 0.1
Gradient index		2.6 ± 0.1
Prescription dose (50% margin, Gy)		5.8 ± 1.1
Number of fractionation		4.0 ± 1.1
BED_10_ (Gy)		3.9 ± 5.8
Diagnosis (lung/breast/others)		22/5/1
Initial tumor volume (cc)		14.0 ± 5.3
Tumor volume (cc) after hfGKRS		9.0 ± 4.3
Volume reduction (%)		96%
Radiation response (complete/partial/progression)		26/16/2

BED = biologically effective dose, hfGKRS = hypofractionated gamma knife radiosurgery, KPS = Karnofsky performance scale, RPA = recursive partitioning analysis.

### 3.2. Planning for gamma knife radiosurgery from LQ model

**Figure 2.**
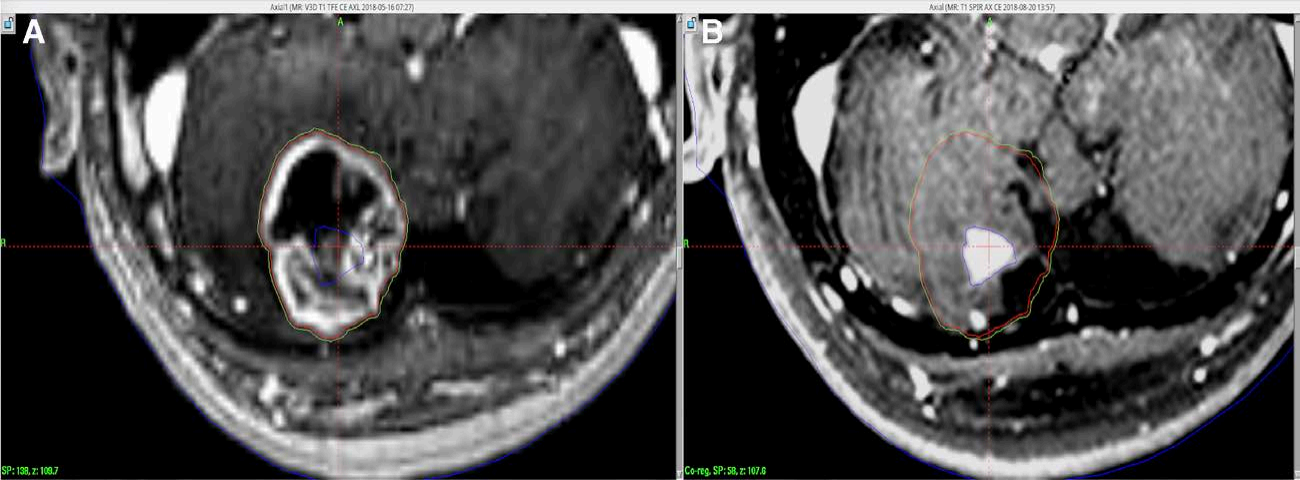
Follow-up MRI of case 1 after hypofractionated gamma knife radiosurgery. Pre (A) and post (B)-operative axial T1-weighted image. Contouring a tumor in red, margin in green. The tumor in blue was decreased at 3 months after hypofractionated gamma knife radiosurgery (follow-up MRI).

**Figure 3.**
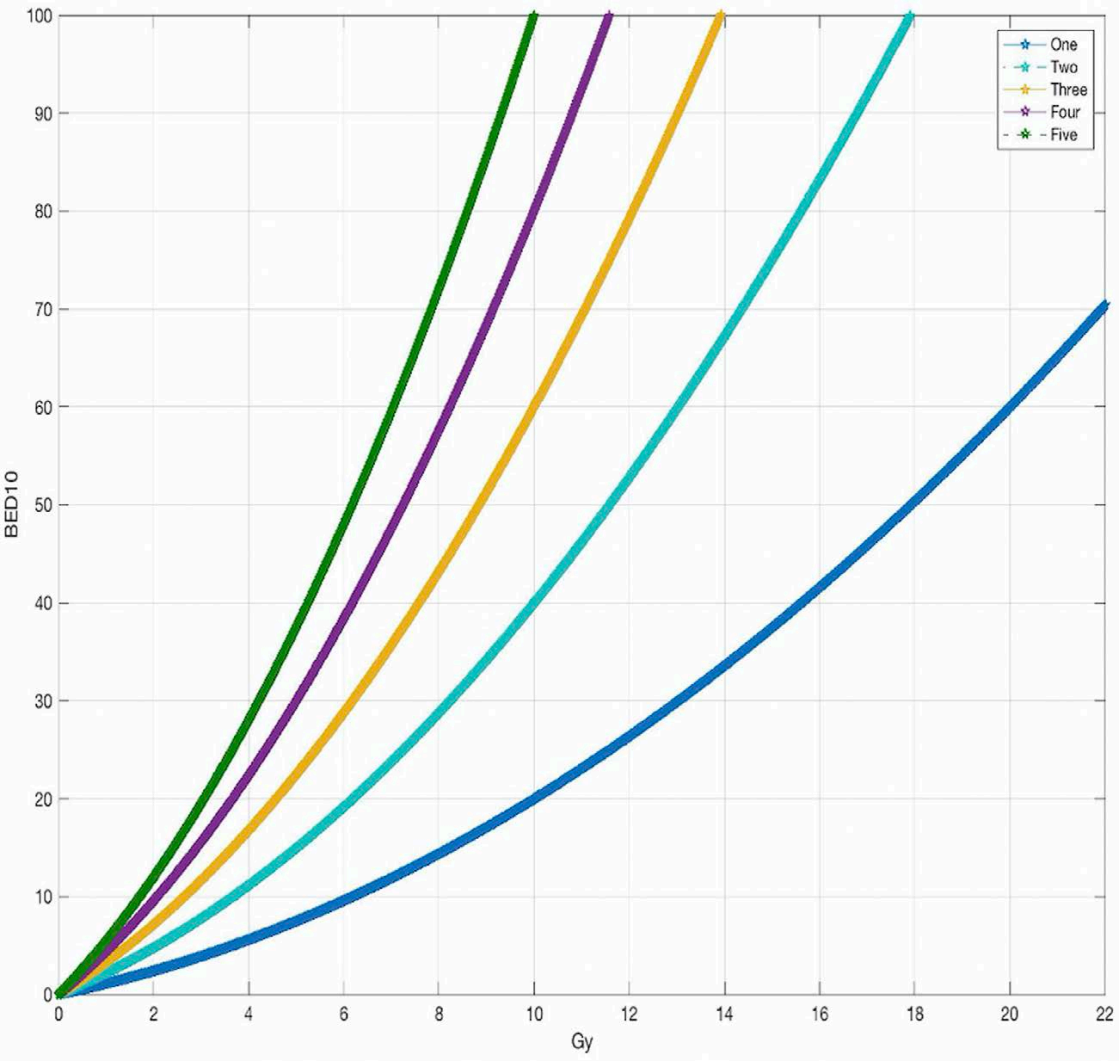
Illustration of biological effective dose_10_ according to the number of fractionated from the linear quadratic model.

When planning for GKRS, the tumor margin was 0.5 mm greater than the existing tumor margin, as shown in Figure 2. We calculated simply the prescription dose (50% margin) from LQ model using MATLAB (Version R2018a, MathWorks, USA). Almost all BM follows an α/β = 10. According to the LQ model equation (BED = nD [1 + [D/ (α/β]]), we chose the prescription dose according to the number of fractions in Figure 3.

### 3.3. Accuracy of predicting volume changes after hfGKRS using ANN

To calculate accuracy, the size of the brain tumor size was converted to an integer for use to achieve the best selection performance. In the test set, the MLP showed an accuracy of 80% and SLP showed an accuracy of 75% for predicting volume changes.

### 3.4. Clinical observations

The follow-up period was from 3 months to 12 months. The local tumor control rate was 96%, and no new metastatic brain lesions were found in all patients. The median overall survival was 6 months. The clinical course of the neurologic deficits included headache (n = 3), motor function deficits (n = 1), and vomiting (n = 1). The follow-up MRI data showed that all patients had improvements.

## 4. Discussion

This study was designed to investigate volume changes after hfGKRS for large BM and predict the volume changes using ANN. The main findings of our case study are as follows: LQ model could be applied to hfGKRS, hfGKRS for large BM reduces tumor size, ANN could predict volume changes with 80% accuracy.

The ANN is the most popular artificial intelligence technique in medical fields. ANNs have been used for clinical diagnosis, image analysis in radiology, data interpretation, neuro-oncology, and histopathology.^[19]^ ANN has similar abilities to computers, can gather and process many variables, and has the capability to be trained by trial-and-error. Therefore, computers could learn to recognize patterns and make informed decisions. This technology is called artificial intelligence and uses variable technology in the medical field.^[20–22]^ However, ANN does not support unique solutions because the trained resting state is based on several factors, including weights, number of cases, and testing cycles. Thus, for certain applications, such as cancer prediction, the frequency distribution of the network versus the outcome probability can be generated, and a central trend including the average, mode, variance measurement, and nonparametric prediction intervals (for a nonparametric distribution with slope) can be created.^[20]^

The LQ model was used to describe the cell survival curve, which consists of 2 mechanisms of cell death by radiation. The purpose of hfGKRS is to deliver an optimized dose to large-volume metastases, as opposed to conventional radiation administration, while minimizing damage to normal tissue. Iwata et al suggested that the LQ formalism has led to incorrect hypofractionated radiotherapy models because of hypofractionated efficacy,^[23,24]^ which is approximately 15%. Additionally, the α/β ratio for metastatic brain tumors is assumed to be 10 to 20, and a higher α/β ratio indicates more sensitivity to fractionated treatments.^[9,25,26]^ The clinical outcomes have not been optimized for hfGKRS for metastatic brain tumors using the LQ model. We found that MLP could predict the clinical outcomes at approximately 80% accuracy. These authors suggested that ANN could be an alternative optimized treatment planning method for predicting clinical outcomes.

We approached the daily fractionated treatment schedule. The daily fractionated treatment has been reported a few. Shoji et al performed 20 to 30Gy given in two fractions 3 to 4 weeks.^[11]^ Kim et al performed fractionated treatment 5 to 11Gy for three to four consecutive days with frame.^[9]^ Dohm et al 15Gy/1fx followed a month later by 14Gy/1fx.^[27]^ The purpose of these strategies with interval time reduced the tumor size after that second re-planning for a smaller volume. In our current study, the volume reduction was 96% and no showed radiation necrosis. We thought that the daily treatment schedule is efficient for large BM.

The present study has some limitations. First, the number of patients with large brain tumors was relatively small, and more samples are needed in future studies. Second, this study showed a cross-section of brain tumor development. Third, the radiation responses by primary cancer type were not compared.

## 5. Conclusions

We analyzed the effect of hfGKRS for large BM and predicted the volume changes through the ANN. As a result, it was shown that hfGKRS from LQ model is effective to apply for large BM. Through our ANN model, it was possible to predict volume changes for large BM after hfGKRS.
